# Supplementary material for: Characterization of a strong covalent Th3+–Th3+ bond inside an Ih(7)-C80 fullerene cage
Source: Nat Commun. 2021 Apr 22;12:2372. doi: 10.1038/s41467-021-22659-2 (PMC8062539; doi:10.1038/s41467-021-22659-2)
Supplement: Supplementary file 1 — Supplementary Information [file 41467_2021_22659_MOESM1_ESM.pdf]

Supplementary Information for

Characterization of a strong covalent Th^{3+}-Th^{3+} bond inside an $I_h(7)$-C_{80} fullerene cage

Authors: Jiaxin Zhuang,[1†] Roser Morales-Martínez,[2†] Jiangwei Zhang,[3†] Yaofeng Wang,[1†] Yang-Rong Yao,[4] Cuiying Pei,[5] Antonio Rodríguez-Fortea,[2] Shuao Wang,[6] Luis Echegoyen,[4] Coen de Graaf,[2,7] Josep M. Poblet,[2*] and Ning Chen[1*]

Affiliations:

1. College of Chemistry, Chemical Engineering and Materials Science, and State Key Laboratory of Radiation Medicine and Protection, Soochow University, Suzhou, Jiangsu 215123, P.R. China.
2. Departament de Química Física i Inorgànica, Universitat Rovira i Virgili, c/Marcel·lí Domingo 1, 43007 Tarragona, Spain.
3. State Key Laboratory of Catalysis, Dalian Institute of Chemical Physics, Chinese Academy of Sciences (CAS) Dalian 116023, P. R. China
4. Department of Chemistry, University of Texas at El Paso, 500 W University Avenue, El Paso, Texas 79968, United States.
5. Center for High Pressure Science and Technology Advanced Research, 1690 Cailun Road, Pudong District, Shanghai, 201203, P.R. China.
6. State Key Laboratory of Radiation Medicine and Protection, School for Radiological and Interdisciplinary Sciences (RAD-X), and Collaborative Innovation Center of Radiation Medicine of Jiangsu Higher Education Institutions, Soochow University, Suzhou, 215123, P.R. China.
7. ICREA, Pg. Lluis Companys 23, 08010 Barcelona, Catalonia (Spain)

*Correspondence to: chenning@suda.edu.cn; josepmaria.poblet@urv.cat.

†These authors contributed equally

This PDF file includes:

Supplementary Discussion

High-performance liquid chromatography (HPLC) separation process of $Th_2@I_h(7)$-C_{80}.

Mass, UV-vis-NIR, Raman and NMR spectroscopies.

X-ray Absorption Spectroscopy (XAS) measurement and analysis.

Supplementary Figures

Fig. 1. HPLC chromatogram and mass spectrum of purified $Th_2@C_{80}$. P6
Fig. 2. HPLC isolation scheme of $Th_2@C_{80}$. P7
Fig. 3. X-ray structure of $Th_2@I_h(7)$-C_{80} showing all identified Th sites. P8
Fig. 4. Fragment view of the interaction of Th1 and Th2 with the closest aromatic ring centers of the cage. P9
Fig. 5. UV-vis-NIR spectrum of $Th_2@C_{80}$. P10
Fig. 6. Atomic orbital overlap as a function of Th···Th separation. P11
Fig. 7. Relationship between $I_h(7)$-C_{80} and $D_2(821)$-C_{104}, views of the computed $Th_2@D_2(821)$-C_{104} and representation of orbitals and electron populations for the ground state of $Th_2@D_2(821)$-C_{104}. P12
Fig. 8. The first derivative of normalized XANES μ(E) spectra of $Th_2@C_{80}$ and ThO_2. P13
Fig. 9. ^{13}C NMR spectrum of $Th_2@I_h(7)$-C_{80}. P13
Fig. 10. Radial distance $\chi(R)$ space spectra of Th in $Th_2@C_{80}$ with k-weight of 3. P14
Fig. 11. Radial distance $\chi(R)$ space spectra of Th in ThO_2 with k-weight of 2. P14
Fig. 12. The relationship between Th and the second coordination shell of the cage and the related Th-C distances. P15
Fig. 13. The relationship between Th and the third coordination shell of the cage and the related Th-C distances. P16

Supplementary Tables

Table 1. Occupancies of disordered thorium sites in $Th_2@I_h(7)$-C_{80}. P17
Table 2. Closest Th-Cage contacts in $Th_2@I_h(7)$-C_{80}. P17
Table 3. Metal−metal distance of dimetallic EMFs with C_{80} or $C_{79}N$ cage. P17
Table 4. Th-Th distances and average Th-Cage distances for experimental and computed $Th_2@I_h(7)$-C_{80} P18
Table 5. Relative energies for $Th_2@I_h(7)$-C_{80} computed at DFT level. P18
Table 6. Electron density topological properties computed for several EMFs. P18
Table 7. Encapsulation energies (or B.E.) in eV computed for several endohedral metallofullerenes at PBE and PBE0 (in parenthesis) levels P19
Table 8. Structural parameters extracted from the L_3-edge Th $\chi(R)$ space spectra fitting of $Th_2@C_{80}$. P19
References P20

Supplementary Discussion

High-performance liquid chromatography (HPLC) separation process of $Th_2@C_{80}$.

The first stage was performed on a BPM column (25 mm × 250 mm, Cosmosil Nacalai Tesque) with toluene as mobile phase. After that, as shown in Supplementary Fig. 2, fraction from 51 to 54 min (marked in blue) was collected and re-injected into a BPD column (10 mm × 250 mm, Cosmosil Nacalai Tesque) for the second stage separation using toluene as the eluent. The fraction marked in orange, which contained $Th_2@C_{80}$ was collected. The third stage of separation was conducted on a BP column (10 mm × 250 mm, Cosmosil Nacalai Tesque) using toluene as the eluent. The fraction marked in green, which contained $Th_2@C_{80}$ was collected and re-injected into the BPD column with a recycle method in the fourth stage. The fraction marked in purple contained $Th_2@C_{80}$ and only a small amount of impurities. The purity of the isolated $Th_2@C_{80}$ was then reconfirmed by chromatography on a Buckyprep column (10 mm × 250 mm, Cosmosil Nacalai Tesque) with toluene at a flow rate of 4.0 mL/min, along with the MALDI-TOF mass spectrometry in a positively charged mode (Supplementary Fig. 1).

Mass, UV-vis-NIR, Raman and NMR spectroscopies

The positive-ion mode matrix-assisted laser desorption/ionization time-of-flight (MALDI-TOF) (Bruker, Germany) was employed for the mass characterization. The UV-vis-NIR spectrum of the purified $Th_2@C_{80}$ was measured in CS_2 solution with a Cary 5000 UV-vis-NIR spectrophotometer (Agilent, USA). The Raman spectrum was obtained using a Horiba Lab RAM HR Evolution Raman spectrometer using a laser at 633 nm. For the Raman measurements, the sample was drop-coated on a quartz plate and the residual CS_2 was removed in a drying chamber in vacuum at 40℃. For the ^{13}C NMR spectroscopic measurements, the $Th_2@C_{80}$ sample was dissolved in CS_2 and placed into the NMR tube. A capillary containing acetone-D6 was used as an internal lock. The ^{13}C NMR spectroscopic measurements were performed at 150 MHz (chemical shift measured in the range of -18 to 220 ppm) with an Avance Ⅲ 600 MHz spectrometer (Bruker, Germany) at 298 K.

X-ray Absorption Spectroscopy (XAS) measurement and analysis

The X-ray absorption spectroscopy (Th L_3-edge) were collected in fluorescence mode using a Lytle detector at BL11B in Shanghai Synchrotron Radiation Facility (SSRF). The samples were filled into the hole with 1.5 mm diameter on PTFE films (film thickness ca. 0.2 mm) for test. Presented data was produced as an average of three consecutive scans for $Th_2@C_{80}$ and two consecutive scans for ThO_2.

XAFS analysis and results: The acquired XAS data were processed according to the standard procedures using the ATHENA module of Demeter software packages.

The EXAFS spectra were obtained by subtracting the post-edge background from the overall absorption and then normalized with respect to the edge-jump step. Subsequently, the $\chi(\mathbf{k})$ data were Fourier transformed to real (R) space using a Hanning windows (dk = 1.0 Å^{-1}) to separate the EXAFS contributions from different coordination shells. To obtain the quantitative structural parameters around central atoms, least-squares curve parameter fitting was performed using the ARTEMIS module of Demeter software packages.

The following EXAFS equation was used:

$$\chi(k) = \sum_j \frac{N_j S_0^2 F_j(k)}{kR_j^2} \cdot \exp\,[-2k^2\sigma_j^2] \cdot \exp\,[\frac{-2R_j}{\lambda(k)}] \cdot \sin\,[2kR_j + \phi_j(k)] \quad (1)$$

The theoretical scattering amplitudes, phase shifts and the photoelectron mean free path for all paths were calculated. S_0^2 is the amplitude reduction factor, $F_j(k)$ is the effective curved-wave backscattering amplitude, N_j is the number of neighbors in the jth atomic shell, R_j is the distance between the X-ray absorbing central atom and the atoms in the jth atomic shell (backscatterer), λ is the mean free path in Å, $\phi_j(k)$ is the phase shift (including the phase shift for each shell and the total central atom phase shift), σ_j is the Debye-Waller parameter of the jth atomic shell (variation of distances around the average R_j). The functions $F_j(k)$, λ and $\phi_j(k)$ were calculated with the ab initio code FEFF8.2. The additional details for EXAFS simulations are given below.

All fits were performed in the radial structure function with *k*-weight of 2. Note that k^2-weight $\chi(R)$ used here would make the scattering path of Th-C bond interaction from second to third coordination shell more recognizable though k^3-weight is commonly

used in the EXAFS analysis and usually provides better resolution. For comparison, the k^3-weight χ(R) have been presented in Supplementary Fig. 10. During the fitting process, the S_0^2, internal atomic distances R, Debye-Waller factor σ^2, and the edge-energy shift Δ were allowed to run freely.

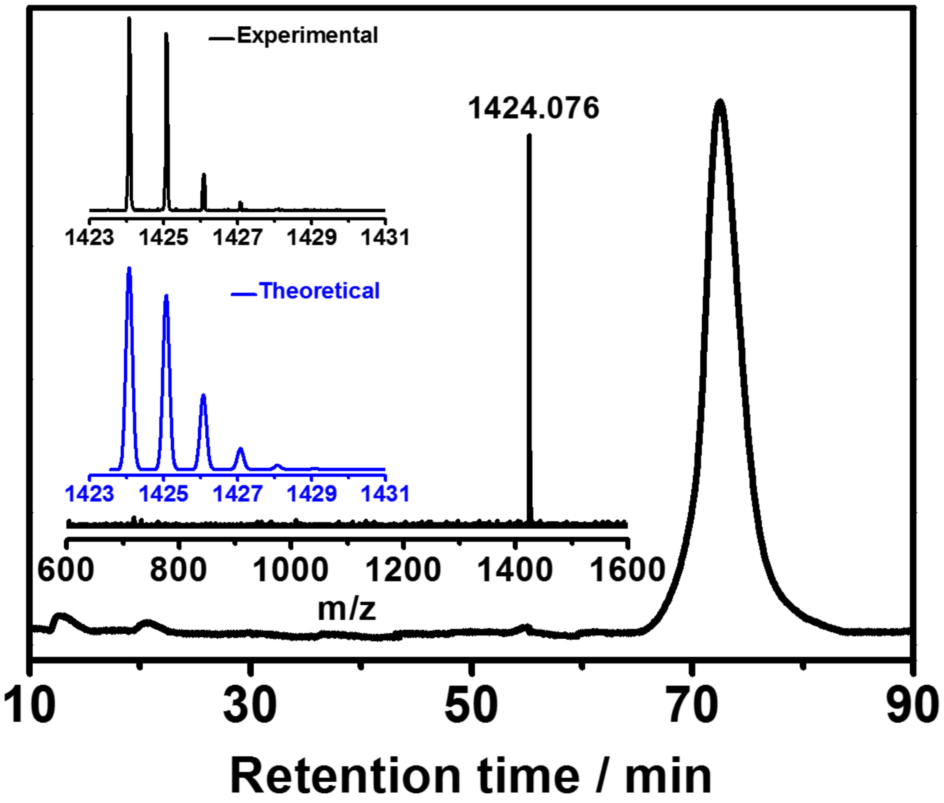

Supplementary Fig. 1. HPLC chromatogram and positive-ion mode MALDI-TOF mass spectrum of purified $Th_2@C_{80}$. Abscissa = Mass-number / Charge-number. Inset: experimental *vs.* theoretical isotopic distribution.

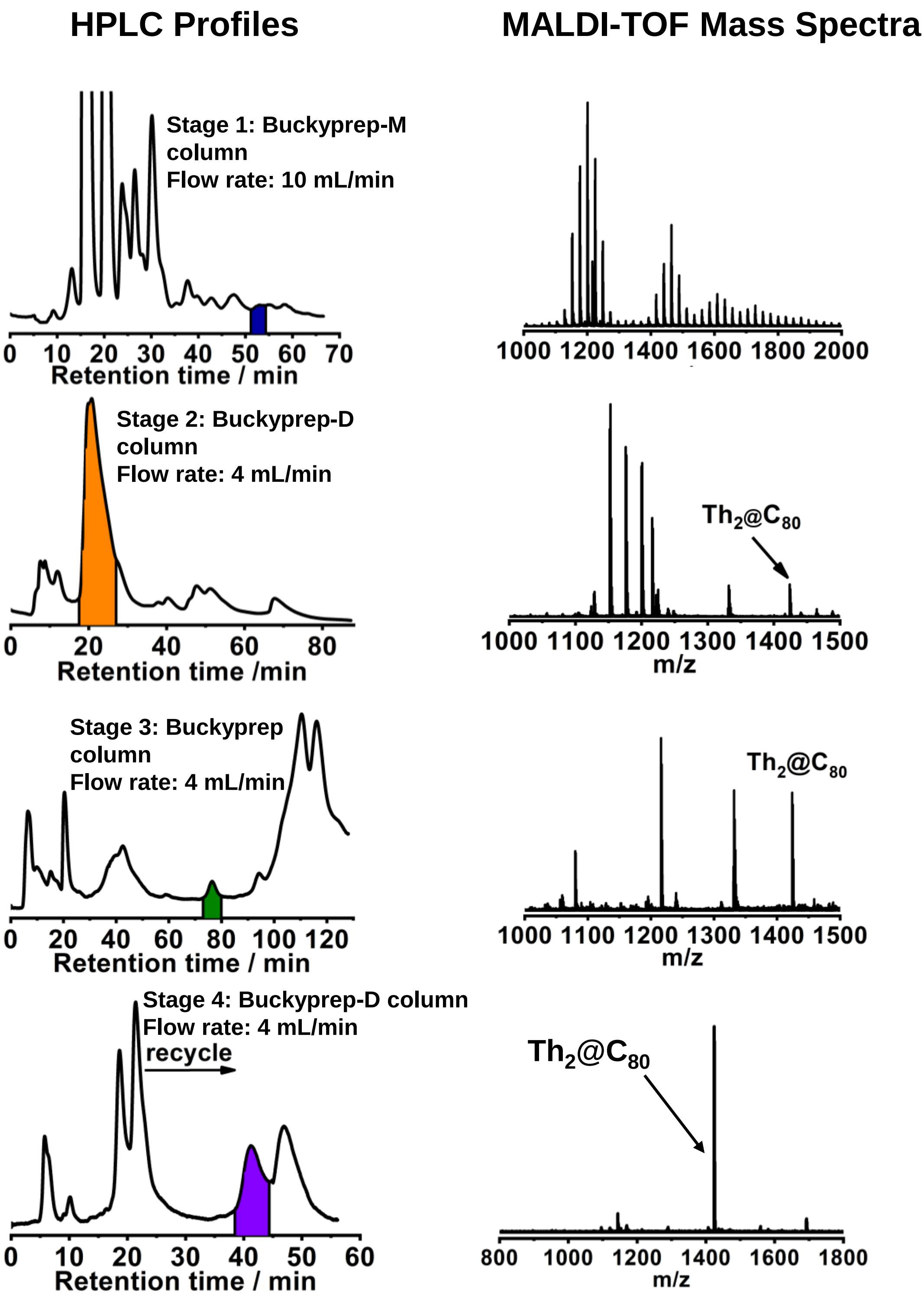

Supplementary Fig. 2. HPLC profiles showing the separation procedures of $Th_2@C_{80}$ (left column) and the corresponding MALDI-TOF mass spectra (right column).

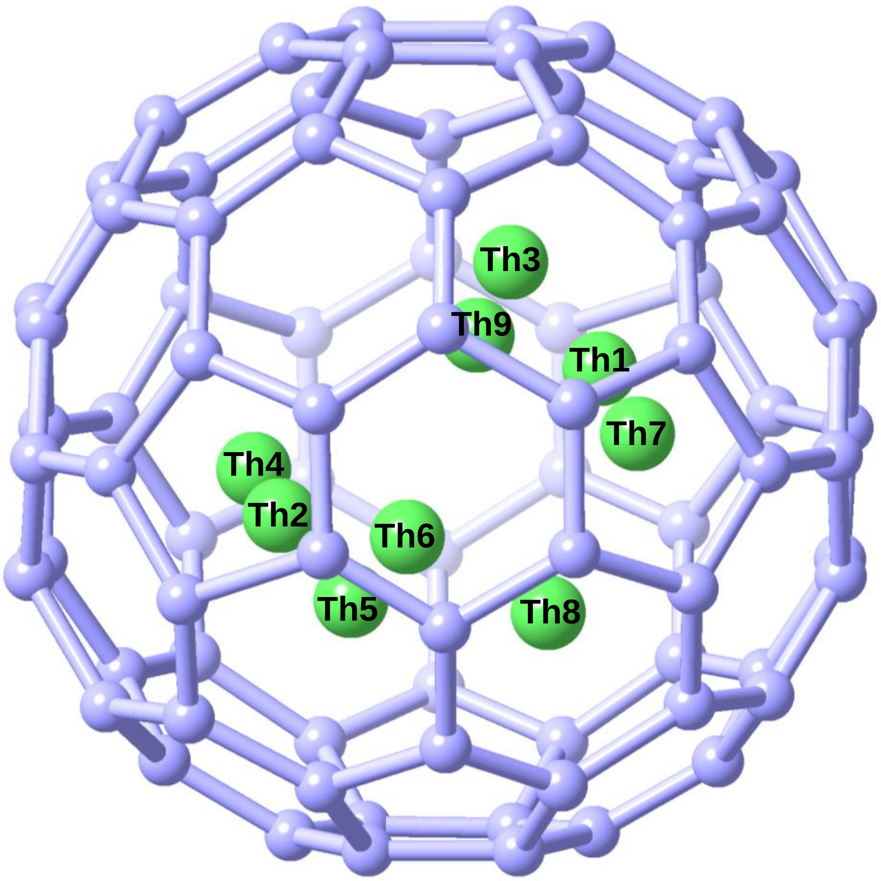

Supplementary Fig. 3. Perspective drawing shows nine positions (Th1-Th9) of the disordered thorium sites in $Th_2@C_{80}$.

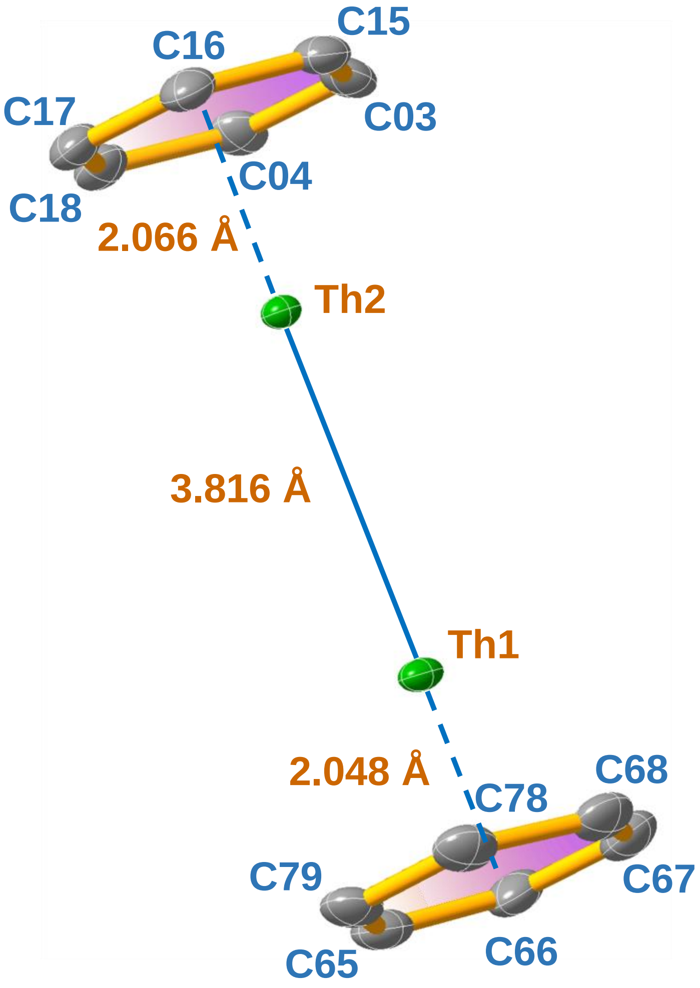

Supplementary Fig. 4. Fragment view showing the interaction of the major Th1 and Th2 cluster with the closest aromatic ring centers fragments of the cage.

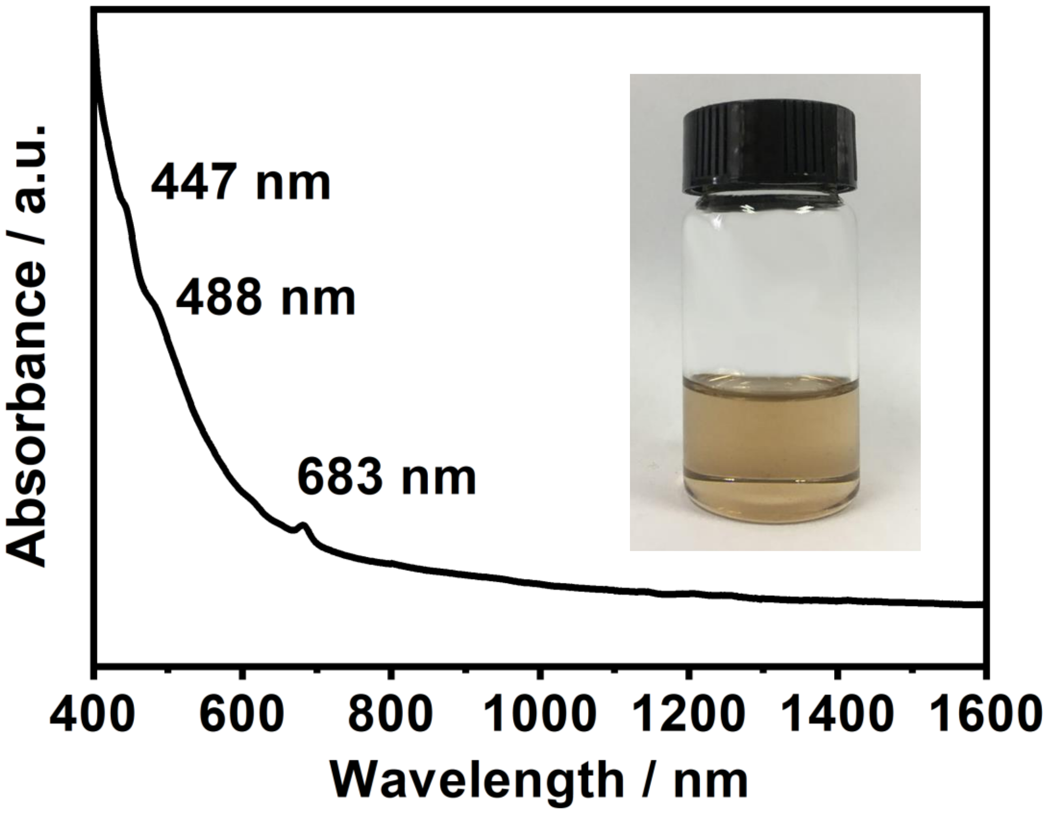

Supplementary Fig. 5. UV-vis-NIR spectrum of $Th_2@I_h(7)\text{-}C_{80}$ in CS_2. The inset shows the photographs of 0.4 mg $Th_2@I_h(7)\text{-}C_{80}$ dissolved in 8 ml toluene.

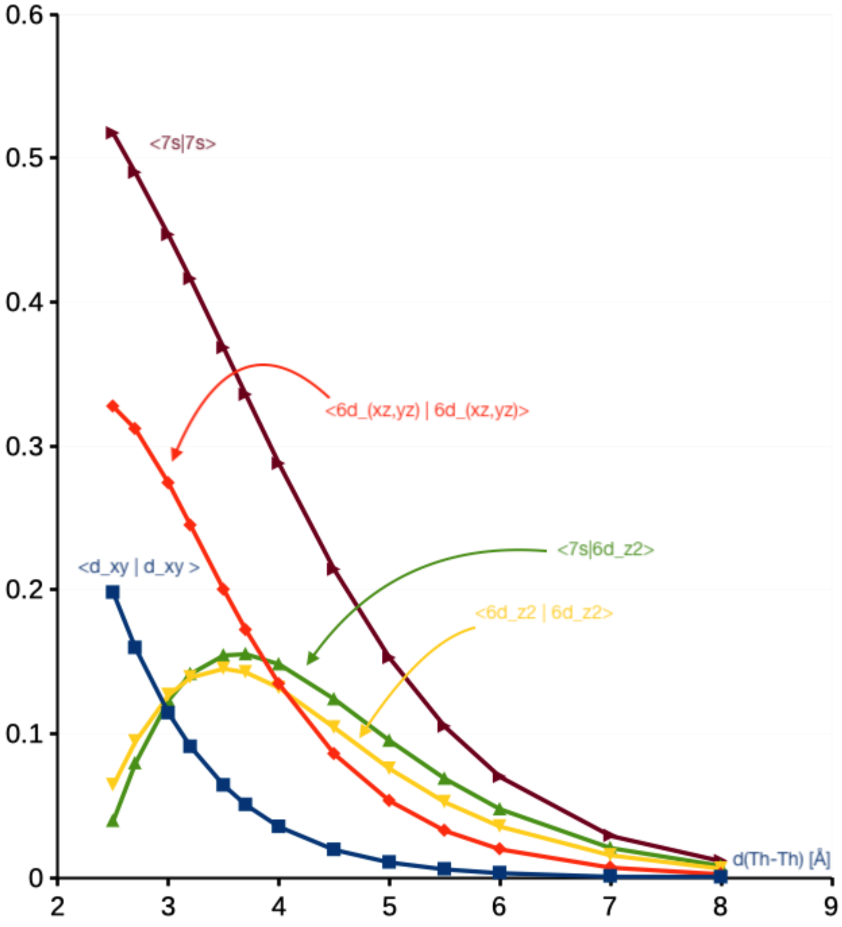

Supplementary Fig. 6. Atomic orbital overlap as a function of Th···Th separation (in Å)

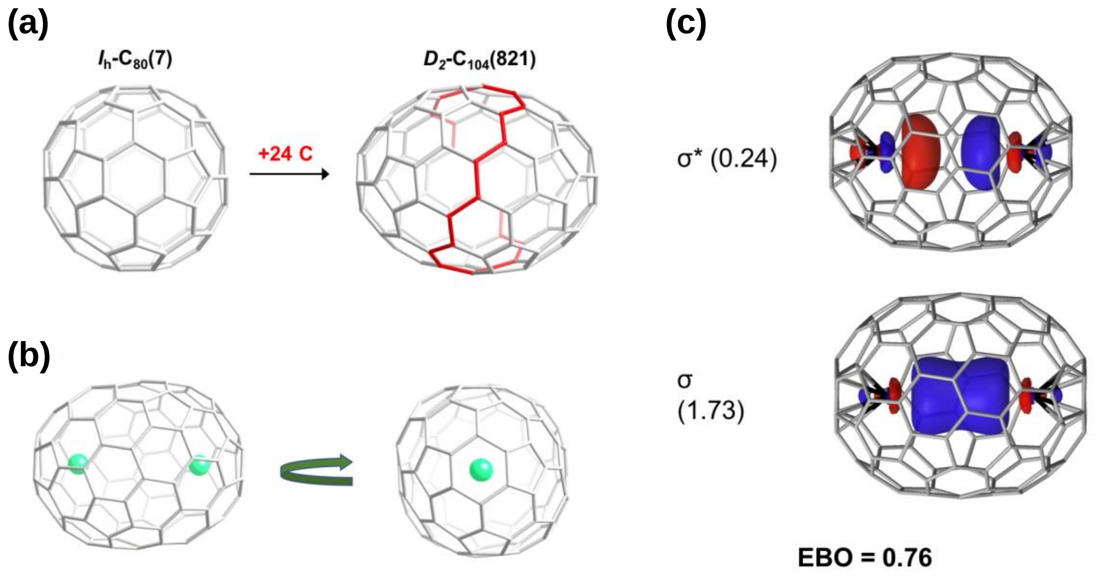

Supplementary Fig. 7. (a) Cage D_2(821)-C_{104} can be seen as the icosahedral C_{80} cage in which 24 atoms have been added in the middle of the six-membered carbon ring. (b) Two views of the computed Th_2@D_2(821)-C_{104} geometry and (c) 3D representation of σ and σ* CASSCF active orbitals as well as electron populations for the ground state of Th_2@D_2(821)-C_{104}, which give an effective bon order of 0.76 at Th-Th bond length of 6.104 Å.

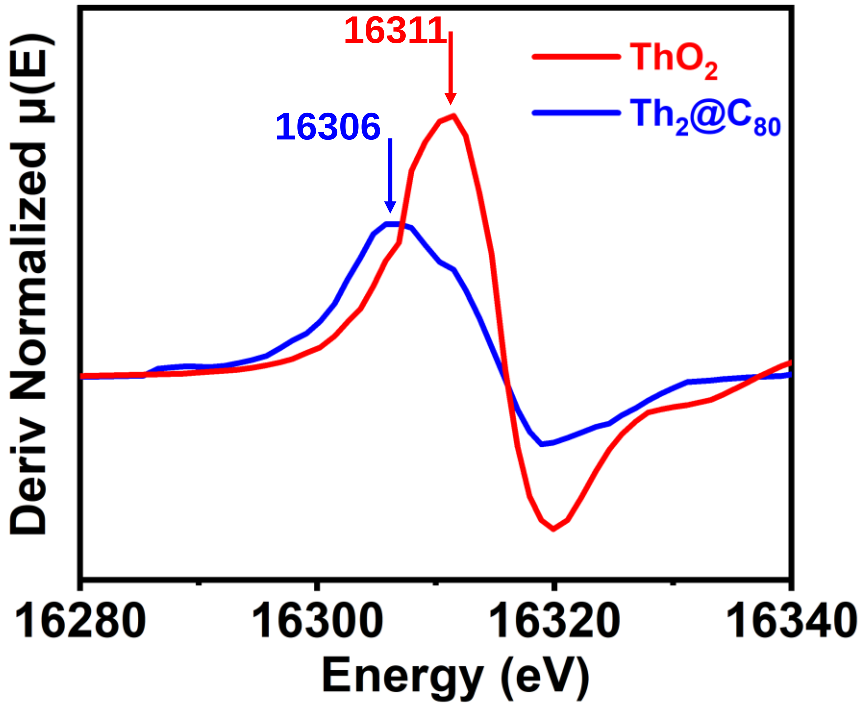

Supplementary Fig. 8. The first derivative of normalized X-ray absorption near edge structure (XANES) μ(E) spectra of Th_2@C_{80} and ThO_2.

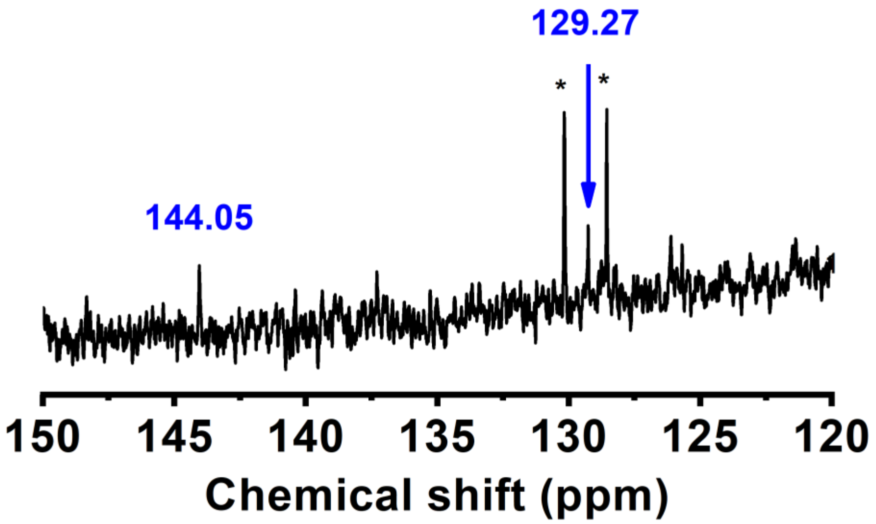

Supplementary Fig. 9. ^{13}C NMR spectrum of $Th_2@I_h(7)$-C_{80} (CS_2, 298 K). A capillary tube containing acetone-d_6 was used as an internal lock. * indicate the contamination by benzene solvent.

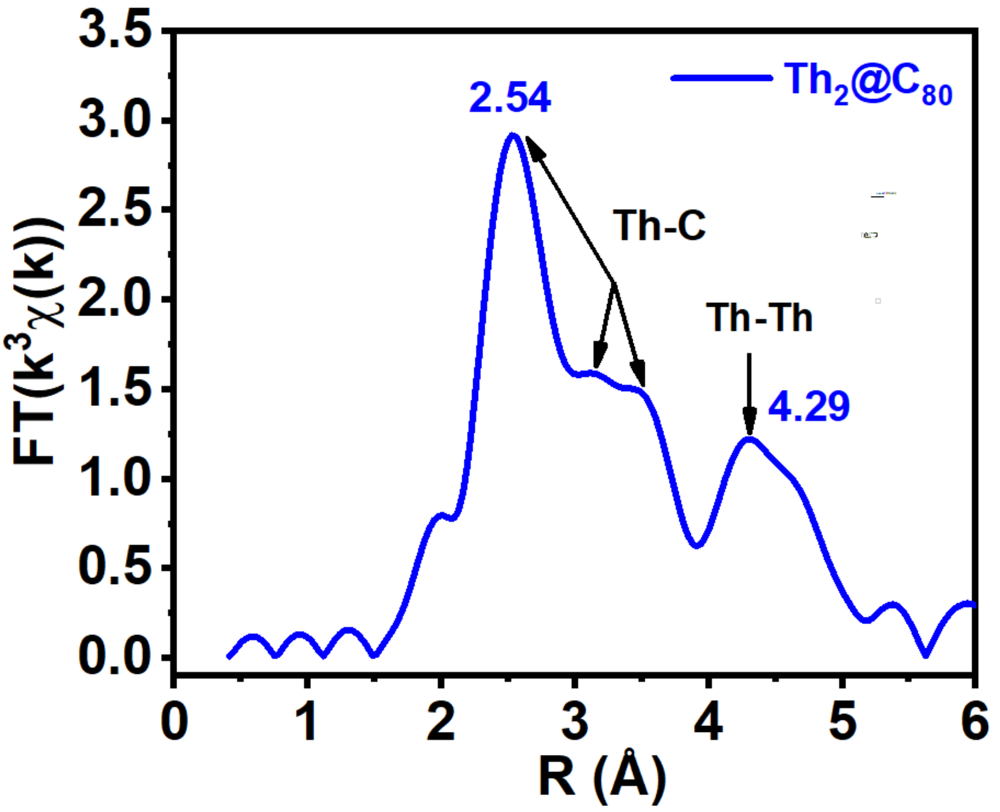

Supplementary Fig. 10. Radial distribution function obtained by Fourier transform of k^3-weighted Th L_3-edge EXAFS spectrum of $Th_2@C_{80}$ measured at room temperature.

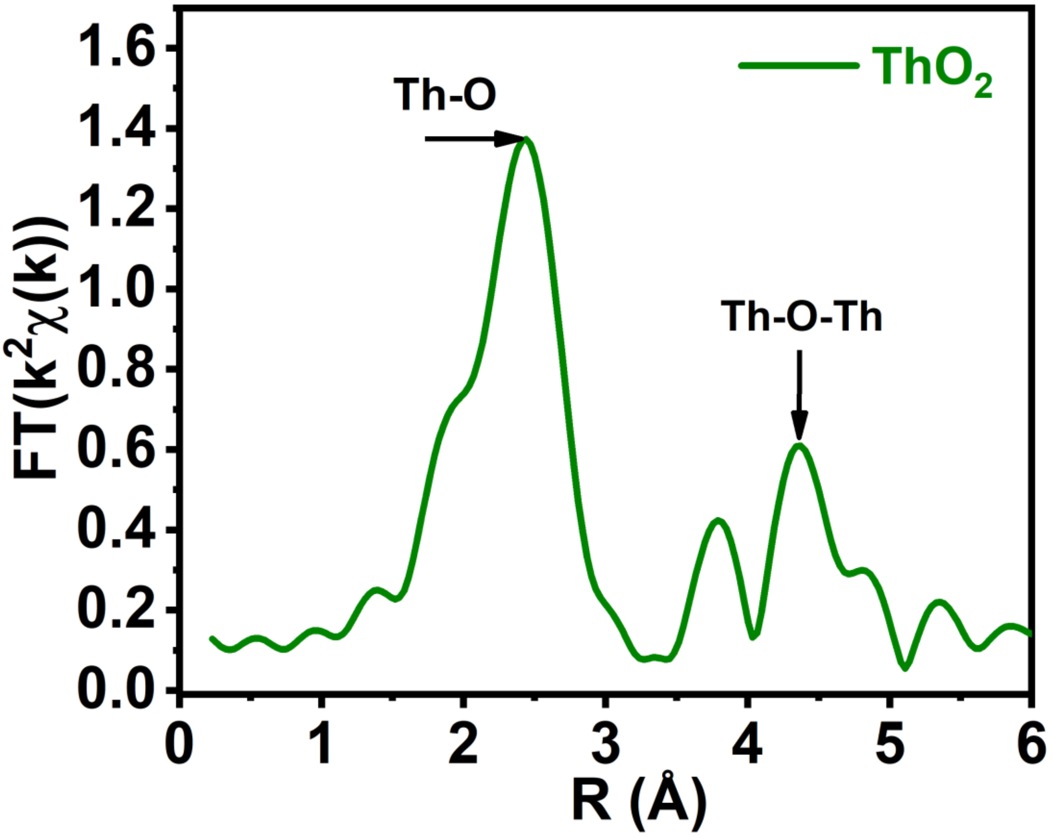

Supplementary Fig. 11. Radial distribution function obtained by Fourier transform of k^2-weighted Th L_3-edge EXAFS spectrum of ThO_2 measured at room temperature.

(a) (b)

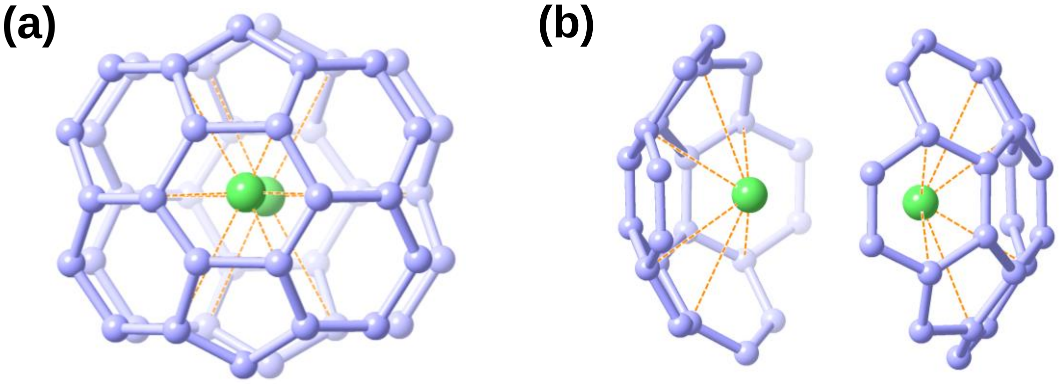

(c)

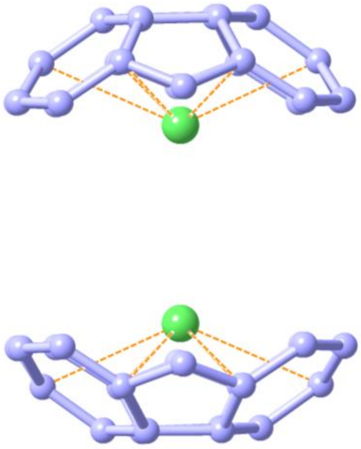

(d)

Th1-C distance (Å)	Th2-C distance (Å)
**Th1-C46 (2.873)**	**Th2-C02 (2.814)**
**Th1-C48 (2.909)**	**Th2-C05 (2.808)**
**Th1-C64 (2.942)**	**Th2-C14 (2.934)**
**Th1-C69 (2.929)**	**Th2-C19 (2.928)**
**Th1-C77 (2.870)**	**Th2-C35 (2.932)**
**Th1-C80 (2.865)**	**Th2-C37 (2.960)**

Supplementary Fig. 12. (a)-(c) Three views of the relationship between Th and the second coordination shell (6 carbons on the periphery of the central hexagon carbon ring) on the fullerene cage of $Th_2@I_h(7)$-C_{80}. (d) Related Th-Carbon distances. (Data from the single crystal CIF file)

(a)

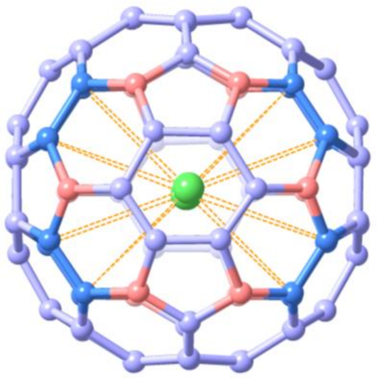

(b)

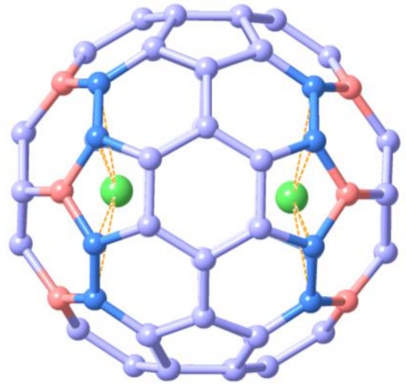

(c)

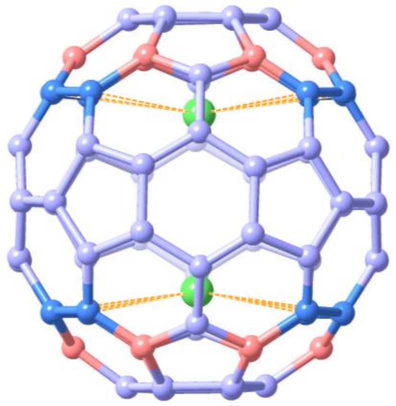

(d)

Th1-C distance (Å)	Th2-C distance (Å)
Th1-C44 (3.369)	Th2-C06 (3.281)
Th1-C45 (3.340)	Th2-C12 (3.309)
Th1-C49 (3.350)	Th2-C13 (3.351)
Th1-C50 (3.350)	Th2-C20 (3.332)
Th1-C62 (3.368)	Th2-C33 (3.390)
Th1-C63 (3.386)	Th2-C34 (3.391)
Th1-C70 (3.355)	Th2-C38 (3.379)
Th1-C71 (3.332)	Th2-C39 (3.379)

Supplementary Fig. 13. (a)-(c) Three views of the relationship between Th and the third coordination shell (8 carbons on the periphery of the second shell 6 carbons) on the fullerene cage of $Th_2@I_h(7)\text{-}C_{80}$. (d) Related Th-Carbon distances. (Data from the single crystal CIF file)

Supplementary Table 1. Occupancies of disordered thorium sites in $Th_2@I_h(7)\text{-}C_{80}$.

**Labelling**	**Th1**	**Th2**	**Th3**	**Th4**	**Th5**
**Occupancy**	0.7830(17)	0.748(2)	0.1299(14)	0.079(2)	0.1283(14)
**Labelling**	**Th6**	**Th7**	**Th8**	**Th9**	
**Occupancy**	0.0508(11)	0.0440(12)	0.0028(7)	0.0342(10)	

Supplementary Table 2. Closest Th-Cage contacts in $Th_2@I_h(7)\text{-}C_{80}$.

**Labelling**	**Th1-C65**	**Th1-C66**	**Th1-C67**	**Th1-C68**	**Th1-C78**	**Th1-C79**	**Th1-ring centroid**
**Length**	2.528(9)	2.506(9)	2.483(9)	2.544(10)	2.503(9)	2.499(9)	2.048
**Labelling**	**Th2-C03**	**Th2-C04**	**Th2-C15**	**Th2-C16**	**Th2-C17**	**Th2-C18**	**Th2-ring centroid**
**Length**	2.484(8)	2.488(8)	2.515(8)	2.540(8)	2.524(9)	2.559(9)	2.066

Supplementary Table 3. Metal−metal distance of dimetallic endohedral fullerenes with C_{80} or $C_{79}N$ cage.

**Compound**	**M−M distance [Å]**	**Ref**
$Th_2@I_h(7)\text{-}C_{80}$	3.816(6)	This work
$U_2@I_h(7)\text{-}C_{80}$	3.793−3.460	Ref 1
$Ce_2@I_h\text{-}C_{80}$	3.833−3.767	Ref 2
$La_2@I_h\text{-}C_{80}$	3.840	Ref 3
$La_2@I_h\text{-}C_{80}(C_7H_7)$	3.779−3.676	Ref 4
$Dy_2@I_h\text{-}C_{80}(CH_2Ph)$	3.896/3.893	Ref 5
$Tb_2@C_{79}N$	3.902	Ref 6

Supplementary Table 4. Th-Th distances and average Th-Cage distances for experimental and computed $Th_2@I_h(7)$-C_{80} with different electronic configurations. [a)]

System	Th−Th distance [Å]	Th−C distance [Å]
experimental	3.816(6)	2.514
σ^2	3.817	2.547
$\sigma^1\ \pi^1$	3.703	2.570
$\pi^1\ \pi^1$	3.508	2.605

a) Calculations at PBE0 level.

Supplementary Table 5. Relative energies for $Th_2@I_h(7)$-C_{80} computed at DFT level.

		BP86		PBE		PBE0	
**Spin state**	**Config.**	$d_{Th\text{-}Th}$ [a)]	ΔE [b)]	$d_{Th\text{-}Th}$ [a)]	ΔE [b)]	$d_{Th\text{-}Th}$ [a)]	ΔE [b)]
singlet	σ^2	3.838	6.1	3.868	4.0	3.817	0.9
triplet	$\sigma^1\ \pi^1$	3.711	0.0	3.737	0.0	3.703	0.0
triplet	$\pi^1\ \pi^1$	3.514	8.5	3.546	10.4	3.508	17.8
triplet	σ^1 cage^1	3.923	11.7	3.951	9.6	3.951	18.7

a) Bond distances in Å; b) Relative energies in kcal·mol^{-1}

Supplementary Table 6. Electron density topological properties computed for several EMFs. [a)]

**Compound**	**Config.**	**M-M** (Å)	ρ_{bcp} [eÅ^{-3}]	$\nabla^2\rho_{bcp}$ [eÅ^{-5}]	
$Th_2@I_h(7)$-C_{80} (D_{2h})	σ^2	3.831	0.234	-0.510	This work
	σ^1cage^1	3.989	0.152	-0.234	This work
$Lu_2@I_h(7)$-C_{80}	σ^2	3.476	0.185	-0.328	Ref 7
$La_2@I_h(7)$-C_{80}^{2-}	σ^2	3.745	0.177	-0.254	Ref 7
$Th_2@D_2(821)$-C_{104} (D_2)	σ^2	6.104	0.032	-0.030	This work
	σ^1cage^1	6.124	0.033	-0.040	This work
$U_2@I_h(7)$-C_{80} (D_{2h})	6e	3.847	0.146	-0.026	This work

a) All values have been obtained from PBE0 molecular orbitals

Supplementary Table 7. Encapsulation energies (B.E.) in eV computed for several endohedral metallofullerenes at PBE and PBE0 (in parenthesis) levels.

Compound	B.E. (eV)[a)]
$Th_2@I_h(7)-C_{80}$ (singlet)	-16.27 (18.72)
$Th@C_1(28324)-C_{80}$	-9.05
$Th@I_h(7)-C_{80}$	-8.36
$La_2@I_h(7)-C_{80}$	-14.57 (16.80)
$La@I_h(7)-C_{80}$	-7.33
$U_2@I_h(7)-C_{80}$	-15.99
$Th_2@D_2(821)-C_{104}$	-14.44 (16.50)
$La_2@D_2(821)-C_{104}$	-14.19 (16.07)

a) Binding energies computed as E(EMF)-(E(cage)+E(nM)); with M = Th or La and n=1 or 2.

Supplementary Table 8. Structural parameters extracted from the L_3-edge Th χ(R) space spectra fitting of $Th_2@C_{80}$.

	Reduced Chi-square (χ_v^2)	R-factor（%）
$Th_2@C_{80}$	592.84	0.0218

amp/ S_0^2	$N_{(Th\text{-}C\ path)}$	$R_{(Th\text{-}C\ path)}$ (Å)	$\sigma^2_{(Th\text{-}C\ path)}$ (10^{-3}Å^2)	ΔE_0 (eV)
1.18+/-0.21	6	2.477±0.127	5.7+/-3.6	2.35+/-1.91
amp/ S_0^2	$N_{(Th\text{-}C\ path)}$	$R_{(Th\text{-}C\ path)}$ (Å)	$\sigma^2_{(Th\text{-}C\ path)}$ (10^{-3}Å^2)	ΔE_0 (eV)
0.81+/-0.16	6	3.100±0.096	5.7+/-3.6	2.35+/-1.91
amp/ S_0^2	$N_{(Th\text{-}C\ path)}$	$R_{(Th\text{-}C\ path)}$ (Å)	$\sigma^2_{(Th\text{-}C\ path)}$ (10^{-3}Å^2)	ΔE_0 (eV)
0.92+/-0.14	8	3.563±0.083	5.7+/-3.6	2.35+/-1.91
amp/ S_0^2	$N_{(Th\text{-}Th\ path)}$	$R_{(Th\text{-}Th\ path)}$ (Å)	$\sigma^2_{(Th\text{-}Th\ path)}$ (10^{-3}Å^2)	ΔE_0 (eV)
0.96 +/-0.18	1	4.241±0.064	7.8+/-4.8	4.83+/-2.27

References

1. Zhang, X.; Wang, Y.; Morales-Martinez, R.; Zhong, J.; de Graaf, C.; Rodriguez-Fortea, A.; Poblet, J. M.; Echegoyen, L.; Feng, L.; Chen, N., $U_2@I_h(7)$-C_{80}: Crystallographic Characterization of a Long-Sought Dimetallic Actinide Endohedral Fullerene. *J. Am. Chem. Soc.* **140**, 3907-3915 (2018).

2. Feng, L.; Suzuki, M.; Mizorogi, N.; Lu, X.; Yamada, M.; Akasaka, T.; Nagase, S., Mapping the metal positions inside spherical C_{80} cages: crystallographic and theoretical studies of $Ce_2@D_{5h}$-C_{80} and $Ce_2@I_h$-C_{80}. *Chem- Eur. J.* **19**, 988-993 (2013).

3. Nishibori, E.; Takata, M.; Sakata, M.; Taninaka, A.; Shinohara, H., Pentagonal-Dodecahedral La_2 Charge Density in [80-I_h]Fullerene: $La_2@C_{80}$. *Angew. Chem. Int. Ed.* **40**, 2998-2999 (2001).

4. Bao, L.; Chen, M.; Pan, C.; Yamaguchi, T.; Kato, T.; Olmstead, M. M.; Balch, A. L.; Akasaka, T.; Lu, X., Crystallographic Evidence for Direct Metal-Metal Bonding in a Stable Open-Shell $La_2@I_h$-C_{80} Derivative. *Angew. Chem.* **55**, 4242-4246 (2016).

5. Liu, F.; Krylov, D. S.; Spree, L.; Avdoshenko, S. M.; Samoylova, N. A.; Rosenkranz, M.; Kostanyan, A.; Greber, T.; Wolter, A. U. B.; Buchner, B.; Popov, A. A., Single molecule magnet with an unpaired electron trapped between two lanthanide ions inside a fullerene. *Nat. Commun.* **8**, 16098 (2017).

6. Zuo, T.; Xu, L.; Beavers, C. M.; Olmstead, M. M.; Fu, W.; Crawford, T. D.; Balch, A. L.; Dorn, H. C., $M_2@C_{79}N$ (M = Y, Tb): Isolation and Characterization of Stable Endohedral Metallofullerenes Exhibiting M−M Bonding Interactions inside Aza[80]fullerene Cages. *J. Am. Chem. Soc.* **130**, 12992-12997 (2008).

7. Popov, A. A.; Avdoshenko, S. M.; Pendas, A. M.; Dunsch, L., Bonding between strongly repulsive metal atoms: an oxymoron made real in a confined space of endohedral metallofullerenes. *Chem. Commun.* **48**, 8031-8050 (2012).
